# Neurodegenerative diseases reflect the reciprocal roles played by retroelements in regulating memory and immunity

**DOI:** 10.3389/fnins.2024.1445540

**Published:** 2024-09-20

**Authors:** Alan Herbert

**Affiliations:** InsideOutBio, Charlestown, MA, United States

**Keywords:** flipons, memory, immunity and antiviral strategies, PKR activation, ADAR1 deaminase, retroelements, virus like capsids, ribotransmitter

## Abstract

Tetrapod endogenous retroelements (ERE) encode proteins that have been exapted to perform many roles in development and also in innate immunity, including GAG (group specific antigen) proteins from the ERE long terminal repeat (LTR) family, some of which can assemble into viral-like capsids (VLCs) and transmit mRNA across synapses. The best characterized member of this family is ARC (activity-regulated cytoskeletal gene), that is involved in memory formation. Other types of EREs, such as LINES and SINES (long and short interspersed elements), have instead been exapted for immune defenses against infectious agents. These immune EREs identify host transcripts by forming the unusual left-handed Z-DNA and Z-RNA conformations to enable self/nonself discrimination. Elevated levels of immune EREs in the brain are associated with neurodegenerative disease. Here I address the question of how pathways based on immune EREs are relate to the memory EREs that mediate neural plasticity. I propose that during infection and in other inflammatory states, ERE encoded GAG capsids deliver interferon-induced immune EREs that rapidly inhibit translation of viral RNAs in the dendritic splines by activation of protein kinase R (PKR). The response limits transmission of viruses and autonomously replicating elements, while protecting bystander cells from stress-induced cell death. Further, the PKR-dependent phosphorylation of proteins, like tau, disrupts the endocytic pathways exploited by viruses to spread to other cells. The responses come at a cost. They impair memory formation and can contribute to pathology by increasing the deposition of amyloid beta.

## Background

**FIGURE 1:**
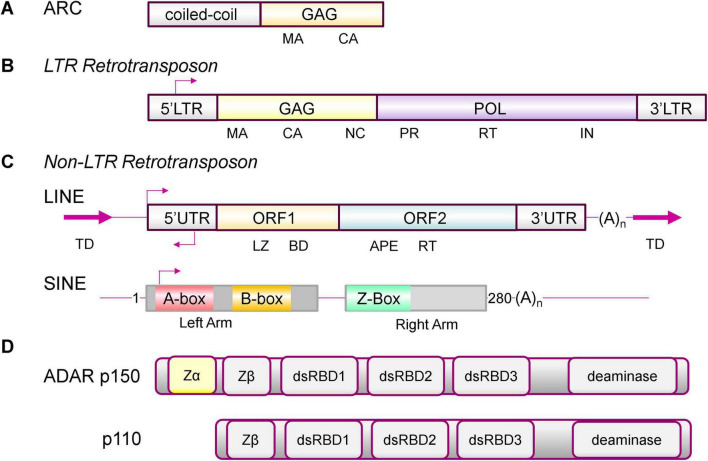
ARC, Endogenous Repeat Elements and ADAR1 isoforms. **(A)** ARC has both the coiled-coil and GAG domains that allow formation of higher order structures, including virus-like-capsids (VLC), but lacks a nucleic acid (NA) binding domain (Ashley et al., 2018; Pastuzyn et al., 2018). **(B)** Long Terminal Repeat (LTR) retrotransposons are composed of a group antigen (GAG) with matrix (MA), capsid (CA) and nucleocapsid (NC) domains, a polymerase (POL) with protease (PR), reverse transcriptase (RT) and an integrase (IN) domain, but lack the envelope protein that is found in retroviruses. The terminal duplication (TD) is of the genomic sequence at the site where the insertion occurred. **(C)** Both LINE (long interspersed elements) and SINE (short interspersed elements) are non-LTR retrotransposons. A full-length LINE has two open reading frames (ORF). ORF1 has a leucine zipper (LZ) a C-terminal nucleic acid binding domain (BD). ORF2 has apurinic/apyrimidinic endonuclease (APE) and a central RT domain. SINEs do not encode proteins. They derive from non-coding RNAs such as 7SL RNA and tRNA. Shown is the dimeric 280 base ALU element with a right and left arm. The A- and B-Box are docking sites for RNA polymerase 3. The Z-Box is a region that can flip to a left-handed Z-DNA conformation under physiological conditions. The Z-box can also form Z-RNA in segments of dsRNA formed by foldback of an ALU inverted repeat (Herbert, 2021). **(D)** The interferon induced ADAR p150 has a Zα domain that recognizes Z-RNA present in double-stranded RNAs. The constitutive p110 isoform does not bind Z-RNA (Herbert, 2021). ZBP1 is the only other protein in the human genome to have a canonical Zα domain (Herbert, 2024b). The arrows indirect the direction of transcription.

**FIGURE 2:**
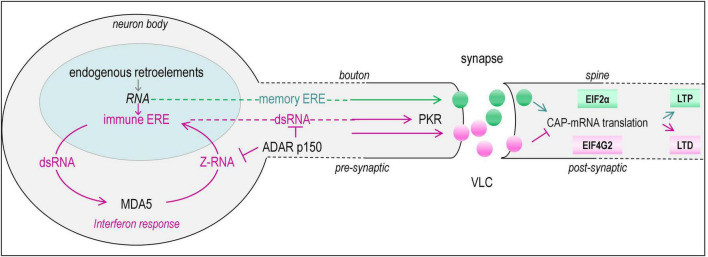
Transmission of RNAs (green spheres) from pre- to postsynaptic neuron is regulated by the retroviral derived GAG proteins and modulated by interferon induced expression of endogenous retro-elements (ERE) (pink spheres) that form double-stranded RNAs (dsRNA). The same coloring of lines is used to highlight the pathways involved. Amplification of the interferon response is driven by MDA5 (melanoma differentiation-associated protein 5, IFIH1, interferon induced with helicase C domain 1) that senses dsRNA. The response is suppressed by the interferon induced p150 isoform of ADAR1 that senses Z-RNA in self-transcripts through a Zα domain and performs A to I editing to trigger degradation of dsRNAs substrates by inosine specific nucleases. A set of GAG proteins encoded by the human genome form VLC (virus-like-capsid), that can incorporate RNA and transmit them to other cells where they are translated. For example, transmission of the memory ERE ARC mRNA by an ARC GAG VLC capsid modulates neuroplasticity. It is proposed here that VLC can also transmit dsRNA ERE transcripts (immune ERE) that enable antiviral defenses in the recipient cell by activating PKR (RNA-activated, encoded in mice by Eif2ak2, eukaryotic translation initiation factor 2 alpha kinase 2). Phosphorylation of EIF2α by PKR causes a switch from CAP-dependent translation to CAP-independent protein production that depends on alternative initiation factors such as EIF4G2. The response promotes local dendritic remodeling and disruption of synaptic contacts to prevent transmission of virus across the synapse and also inhibits the translation of viral messages in the postsynaptic neuron. The changes also protect against stress-induced apoptosis and necroptosis, but impair memory formation by altering the balance of LTP (long term potentiation) to LTD (long-term depression). The dotted lines indicate that the axon is much longer than shown as the figure is not drawn to scale.

Retroviruses and Memory: A recent surprise was the discovery that a retrovirally derived GAG (group associated antigen) protein called ARC (activity-regulated cytoskeletal gene, Figure 1A) was associated with memory formation in tetrapods. Even more surprising was the finding that a similar but separate domestication event of an ARC protein impacted a specific set of feeding behavior in *Drosophila melanogaster*. If that was not enough, two groups then revealed that ARC proteins from human, mouse and flies were able to form viral-like capsids (VLCs) (Epstein and Finkbeiner, 2018; Ashley et al., 2018; Pastuzyn et al., 2018). The Drosophila Arc1 protein also bound *darc1* mRNA in neurons and loaded the transcript into extracellular vesicles that were transferred across the synapse formed between motor neurons and muscles (Ashley et al., 2018). Rat Arc protein assembled *in vitro* in the presence of both *Arc* and enhanced green fluorescent protein (EGFP) RNA into capsids, with little specificity shown. The capsid mediated the transfer of Arc mRNA and EGFP mRNA into new target cells via extracellular vesicles released from neurons (Pastuzyn et al., 2018), and also of luciferase mRNA (de la Pena et al., 2021). The mRNA, referred to here as ribotransmitters, underwent activity-dependent translation in the recipient neuron following uptake by endocytosis (Figure 2) (Pastuzyn et al., 2018), consistent with the proposed role of modulating synaptic connectivity (Wallace et al., 1998).

The tetrapod and *D. melanogaster* ARC proteins are derived from separate branches of the Metaviridae family of long terminal repeat (LTR) endogenous retroelements (ERE) (formerly known as Ty3/Gypsy retrotransposons) (Gifford et al., 2018) (Figure 1). Further, insects are the only protostomes and tetrapods the only deuterostomes that encode ARC, with no other known paralogs that connect to their last common ancestor. The findings support an independent origin of each exaptation (Abrusan et al., 2013). Both tetrapod and insect versions are expressed as neuronal immediate early genes. Whereas the tetrapod version is associated with synaptic plasticity, the insect ARC is not. Rather, the *D. melanogaster* protein increases locomotory activity in response to starvation (Mattaliano et al., 2007). Structural characterization of the drosophila Arc VLC has revealed that the capsid has a number of unique features. While the ancestral Ty3 packages two copies of its 5.2-kb genome into a T = 9 particle, the dArc1 VLC is smaller. At most, it is only able to package two copies of the 2.3-kb dArc1 full length mRNA into its T = 4 structure (Erlendsson et al., 2020). By comparison, the HIV (human immunodeficiency virus) genome is 9.3 kb. The mRNA is also packaged into the capsid as a dimer. The HIV transcript consists of a 1.5 kb GAG, a 2.8 kb POL (polymerase) and a 2.6 kb ENV (envelope) open reading frame (ORF) (Figure 1A). LTRs lack the ENV gene found in retroviruses.

The human genome also encodes other proteins able to assemble into VLC. There are 85 human genes known with homology to retroviral or retrotransposon-encoded GAG genes (Campillos et al., 2006; Kokosar and Kordis, 2013). Of these, eight clades are universally retained as at least single intact genes across all placental mammals queried, suggesting they enable important biological functions common to all mammals (Henriques et al., 2024). These include PEG10 that can accommodate large RNAs and is able to bind and secrete its own 6.7 kb mRNA, as well as another 49 mouse RNAs, which have reduced neurological expression in PEG10 knockout mice, suggesting that PEG10 binds and stabilizes these transcripts. Interestingly, flanking a coding sequence with the PEG10 untranslated region (UTR) is sufficient to enable the inclusion of the RNA construct into the PEG10 VLC. The efficiency of VLC uptake and translation of the message in the recipient cells is further increased by incorporating a fusogen to form a virus like particle. Other exapted murine GAGs such as MOAP1, ZCCHC12, RTL1, PNMA3, PNMA5 and PNMA6a are also known to form capsids, but their properties have not yet been fully characterized (Segel et al., 2021). Of these eight GAG variants, hARC is the oldest, dating back an estimated 350 million years. The next oldest is PEG10 (paternally expressed 10) from 160 million years ago. PEG10 marks the first known appearance of genomic imprinting where the sex of the parent determines which copy of an autosomal gene is expressed. The innovation occurred in placental animals and is implemented by the selective methylation of the PEG10 gene in females (Henriques et al., 2024; Suzuki et al., 2007; Shiura et al., 2023).

ARC is important in the formation of tetrapod memories. It is also likely that PEG10 and other GAG proteins can assemble into capsids and contribute to communication between neurons through the synaptic transmission of RNA (Segel et al., 2021). A number of questions remain unanswered beyond those related to which RNAs, other than ARC, function as ribotransmitters and what are the outcomes they regulate? One important question is how did these innovations evolve? These adaptations require time but these autonomously replicating EREs are invasive and pose an existential threat to their host genome. How were these EREs tamed sufficiently for beneficial outcomes to accrue? What accommodations were made to balance the risks associated with EREs and the benefits from their exaptation? How well do the modern-day defenses against autonomous replication by EREs work? What happens to individuals when these defenses fail?

## Conflicted genomes

The conflict between an organism and its invasive replicants dates back as far as we can tell. There is no doubt that symbiosis between different entities led to early evolutionary innovations where the parts supplied by each genome benefited all. Two extreme, but successful, examples of this confluence are the mergers that generated chloroplasts and mitochondria (Margulis, 1976). Before those events, many unicellular organisms and viruses exchanged genetic information, supplying parts that the other was missing and providing weapons for attacking and defending against other replicants. The horizontal transfer between species of adaptations and virulence factors often led to quite complex survival strategies. The outcomes are strikingly exemplified by modern day amoeba that have incorporated DNA from all the genomes they have ever encountered, and now harbor in a single individual a cohort of viruses, phages, and bacteria pathogenic to humans (Molmeret et al., 2005; Moliner et al., 2010).

The acceptance of foreign DNAs into a genome often confers a benefit. The strategy also carries risk. The threat is especially serious when the newly acquired DNA is capable of autonomous replication, as is the case with transposable elements that can duplicate themselves exponentially within a host genome. The existing and invasive replicants are often at odds. While an expanded genome increases the coding capacity of an existing organism, the invading replicant often prioritizes smaller genome size and the efficient packaging of as many infectious variants as possible. How organisms cope with such different imperatives shapes their future evolution.

The ARC gene is an example of a beneficial acquisition of a retroviral element by tetrapod genomes. Other examples exist. A retroviral ENV gene required for placenta formation has been independently exapted in seven separate species to produce a syncytin protein, illustrating the selective advantage accruing from this innovation (Dupressoir et al., 2012; Imakawa et al., 2022). ERE regulatory elements have also been exapted in zygotes to control the transition from totipotent to pluripotent stem cells, a key step in embryonic development (Chuong, 2018; Macfarlan et al., 2012).

## ERE biology

The GAG proteins like ARC are derived from a LTR retrotransposon (Figure 1B). In humans, LTRs can vary in size from 100 to several kilobases, with strong selection against retention of the POL gene that is necessary for their autonomous replication (Lynch and Tristem, 2003). Additionally, capsids like those formed by ARC are likely too small to incorporate both the GAG and POL transcripts. Other classes of ERE that lack the LTR (called non-LTR transposons) are also present in the human genome (Figure 1C). This family includes the autonomous long interspersed elements (LINEs) and the non-autonomous short interspersed elements (SINEs) (Wessler, 2006). LINES are usually flanked by variable length duplication of sequences from the sites where they insert. They have a promoter at the 5′ end of the element and a short 3′UTR that ends in a poly-A tail. Their transcription depends on RNA polymerase 2 (POL2). The full length bicistronic ∼6 kb RNA they produce encodes two proteins: ORF1 (an RNA binding protein) and ORF2 (an endonuclease and a reverse transcriptase). LINEs date back to the Precambrian era of 600 million years ago, with 4 of the 15 known clades present in mammals. About 18% of the human genome consists of LINE-1 (L1) EREs. There are roughly 500,000 L1 copies, with some of them still capable of autonomous replication (Goodier and Kazazian, 2008). Their regulatory elements have contributed to many evolutionary innovations (Lowe and Haussler, 2012). Remarkably, these elements now help protect their host. They drive the interferon (IFN) induced expression of genes involved in the innate immune responses (Chuong et al., 2016).

SINEs contribute to about 11% of the human genome, with over 1 million copies, but are absent in D. melanogaster (Sela et al., 2010). They are notable because they do not encode protein but instead are derived from POL3 transcribed small, cellular, noncoding RNAs (ncRNA). The most frequent SINE is derived from the 7SL RNA component of the protein signal recognition particle. In humans, they are named ALU elements for the bacterial restriction enzyme that excises them from genomic DNA. Other SINE families originate from tRNAs and less frequently from snoRNAs and other ncRNAs. ALUs are 90 to 300 bases long, depending on whether they are a single or a duplicated copy of the ancestral RNA (Figure 1C). Many ALUs are within genic regions and are now transcribed by POL2 (Deininger, 2011). For their propagation, they capture newly synthesized LINE reverse transcriptases as they emerge from the ribosome. The SINEs then use those enzymes to copy their RNA into DNA. In the process, the new copy is pasted into the genome at a site different from that of the original copy. The freshly inserted SINE can carry with it some regulatory elements from the old ALU neighborhood, altering the readout and processing of RNAs from nearby genes. Unlike mutations, the outcomes are not all or nothing: successful RNA adaptations from the past are not immediately lost, but instead are produced alongside the new transcripts that may differ in their stability, splicing and translation. Malformed pre-mRNAs are purged through processes like nonsense-mediated decay (Herbert, 2020a; Herbert, 2024a). The soft-wiring of RNA processing increases the diversity of protein products produced. Often, SINEs are inserted in the reverse orientation to an existing SINE element. When transcribed, the RNA formed can fold back on itself to produce a double-stranded RNA (dsRNA) (Levanon et al., 2004; Kim et al., 2004; Blow et al., 2004; Athanasiadis et al., 2004). The outcome is common in humans, reflecting the three recent waves of ALU invasion that represented an existential threat to their genome (Herbert, 2020a; Batzer and Deininger, 2002). Those inverted repeats that form dsRNAs are subject to RNA editing by the ADAR (adenosine deaminase, RNA specific) family of enzymes (Schaffer and Levanon, 2021). The enzyme deaminates adenosine in dsRNA to form inosine (A to I editing). Since inosine is translated as guanosine, the readout of adenosine containing codons is potentially altered, resulting in the incorporation of a different amino acid into a protein. The result is a protein isoform that is not templated by the host genome (Higuchi et al., 2000; Maas et al., 1996; Sommer et al., 1991). As we will see, ADAR1 performs other very important roles in defending the host and in forming memories.

## Defending against retroelements

The processes we observe today date from long ago. There was an early need to tame EREs capable of autonomous replication, given the threat they posed to the survival of a species by inserting into and inactivating essential genes. Often, to resolve the conflict, the offensive weapons used by EREs were repurposed to defend against them: a battle of like with like. Often the exapted parts were used to restrict ERE replication. GAG proteins initially used to package EREs into membrane coated vesicles were retooled to disrupt the ERE assembly line. Subsequently, those GAG variants evolved into something else. Some GAGs are now employed as immune sensors in the brain, with RTL5 sensing dsRNA and RTL6 now detecting bacterial lipopolysaccharides (Irie et al., 2022) while RTL9 recognizes fungal zymogens (Ishino et al., 2023). Another strategy was to prevent ERE transcription, so limiting retrotransposon spread. The zinc finger domains encoded by the LTRs to bind their transcripts were repurposed to repress their descendants, leading to clusters of KRAB (Krüppel-associated box) protein families that suppress ERE expression (Ecco et al., 2017). The KRAB genes encode different combinations of the zinc-finger sequences that they have captured and embellished. The clusters potentially produce even more permutations by trans-splicing RNAs from different genes (Umerenkov et al., 2023). The many KRAB variants generated counter any attempted escape by ERE based on the recombination of existing sequences. Such defensive strategies take time to evolve. Before then, organisms required other schemes to ensure their proximate survival.

The destruction of a transposon transcript provides an immediate defense. Mechanisms such as RNA interference enable the instant elimination of autonomously replicating RNAs. Another set of noncoding transcripts, such as microRNAs, prevents the translation of ERE proteins that mediate transposition. Both mechanisms depend on host RNAs complementary to the invasive RNA (Cornec and Poirier, 2023). Indeed, many microRNAs are thought to derive from the dsRNAs formed in the past by retroelement sequences. Later, these microRNAs evolved to play a larger role in host development, targeting the retrotransposons sequences inserted into genes as ERE peppered themselves throughout the genome (Lehnert et al., 2009; Obbard et al., 2009; Borchert et al., 2011).

## Avoiding reverse transcriptases

Many organisms have evolved mechanisms to amplify RNAs specifically targeting transposons like ERE. In many species, this pathway uses an RNA-dependent RNA polymerase (RdRp) to generate the dsRNA substrate necessary to produce a guide RNA (gRNA) that paints the target for the defense to destroy. The RdRps involved are generally not very processive, often not in need of priming and usually do not produce long transcripts (Almeida et al., 2019). This strategy is exemplified by the transitive RNAi of the round worm *Caenorhabditis elegans* (Obbard et al., 2009). Of course, this solution requires a companion strategy to avoid an attack on self-RNAs. In C. elegans, there is a dedicated RNAi pathway that prevents the destruction of germline transcripts, one based on the CSR-1 (chromosome segregation and RNAi deficient 1) protein (Wedeles et al., 2013; Seth et al., 2013). *D. melanogaster* instead uses reverse transcription to make a circular DNA copy of the pathogen to generate the required gRNA with the product disseminated via exosomes (Tassetto et al., 2017). The pathway can produce transgenerational immunity. How this strategy discriminates self from nonself is not yet fully understood (Mondotte et al., 2020). *D. melanogaster* also deploys another strategy to defend against EREs, required as the LTRs in fly genomes are still highly active. Here gRNAs are produced by “ping-pong” amplification of short *piwi* RNAs (piRNA). The gRNAs are defined by the piRNA gene clusters that are expressed mostly in the germline. The mechanism does not require a RdRp (Czech et al., 2018).

Interestingly vertebrates have adopted a different system to protect against autonomous replicants. The response does not rely on amplification of the invader’s RNA, avoiding the risk of promulgating a pathogen by making further copies of its genome. The strategy also avoids the destruction of essential host transcripts containing embedded EREs. Instead, RNAs that incorporate EREs are used to protect the host. These transcripts are often encoded by parts of the genome that are silent most times. The ERE containing sequences are placed beyond the usual transcription stop site of a gene or removed by splicing. However, when cells are infected by viruses, or become dysregulated, these alarm sequences are transcribed or remain unspliced (Jang and Latchman, 1992; Panning and Smiley, 1993; Karijolich et al., 2017; Tucker and Glaunsinger, 2017). The transcripts produced then form dsRNA that are sensed by their shape rather than by their sequence, triggering an innate immune response that terminates the threat.

The strategy traces back to the earliest known unicellular eukaryotic progenitor, *Capsaspora owczarzaki*. In these organisms, invasive replicants that form dsRNA are targeted for A to I editing by the ADAR1 ancestral protein (Sebe-Pedros et al., 2016; Ros-Rocher et al., 2021). Over time, DNA for these non-self dsRNAs have been copied into host genomes. Their transcription was regulated by the same elements that an invasive replicant used to drive the expression of its own genome (Chuong et al., 2016).

In vertebrates, the acquired dsRNAs were exapted to drive the modern-day IFN-induced, antiviral defense. The dsRNAs produced were repurposed to shutdown translation of the invader’s transcripts, preventing their transmission to other cells. Vertebrates evolved PKR (Kinase, RNA-activated, encoded in mice by *Eif2ak2*, eukaryotic translation initiation factor 2 alpha kinase 2) just for this purpose: PKR is both IFN-induced and dsRNA activated (Roberts et al., 1976). As the gene name implies, PKR targeted the EIF2α translation initiation factor. The rapid evolution the PKR gene has undergone in response to viral threats underscores the importance of this innovation (Rothenburg et al., 2009; Bou-Nader et al., 2019; Cesaro and Michiels, 2021). As I will describe, the evolution of PKR also has impacted the development of adaptive memories within the nervous system.

## Self versus Non-self

The dsRNA formed by embedded ERE also enable self/non-self-discrimination without requiring a reverse transcriptase. By flipping from a right-handed A-RNA conformation to left-handed Z-RNA, the ERE dsRNA provides a mechanism for self-recognition, as I will now describe. The Z-DNA is recognized by the IFN the p150 isoform of ADAR. Unlike the constitutively expressed p110 isoform, p150 has a Z-RNA/Z-DNA (collectively called ZNA) binding domain, called Zα, that binds with high affinity the left-handed double helical conformation of both DNA and RNA (Figure 1B). While right-handed B-DNA and dsRNA A-RNA are normally the lowest energy nucleic acid helices found in the cell, both left-handed Z-DNA and Z-RNA (collectively called ZNAs) are also present. Like dsRNA, ZNAs convey information by their shape rather than their sequence. They are higher energy forms of DNA and RNA that are most easily adopted by segments with a dinucleotide repeat composed of alternating purine and pyrimidine bases. Z-DNA is formed more easily by (CG)_*n*_ than (TG)_*n*_ or (AC)_*n*_, while (TA_)n_. is more prone to form hairpins. ZNAs are also formed inside cells by out of alternation sequences like (CCCG)_*n*_ (Ho et al., 1986; Nichols et al., 2021). The transition involves flipping the base pairs over and does not require strand cleavage. The dinucleotide sequence motif results in a zig-zag backbone for which the conformation is named.

The genomic elements that convert to ZNA under physiological conditions. are called flipons (Herbert, 2019a). The flip requires energy that can be supplied by hydrolysis of high energy substrates like ATP or other nucleotides. It is driven by polymerases, helicases and opposed by enzymes like topoisomerases that relax the forces such as twisting, stretching, and bending that power the transition both in the nucleus and in stress granules (Chiang et al., 2020; Yi et al., 2022). By changing conformation, flipons flag actively transcribed genes, serving many roles in a cell (Herbert, 2024a; Herbert, 2023). The sequences are enriched in promoters and also in the repeat part of the genome, especially in EREs, that otherwise have little informational value due to their high frequency. Indeed, one repeat looks very much like another (Herbert, 2023).

Like ADAR1, the Zα domain also traces back to *C. owczarzaki* (Herbert, 2024b). Mounting evidence supports the involvement of Zα in recognition of self-RNAs, with noncoding ALU SINE transcripts being the most frequently edited RNAs in the human transcriptome. Importantly, dsRNAs formed from SINE inverted repeats contain a Z-BOX that forms Z-RNA. The flip to Z-RNA provides a tag to label transcripts as self, allowing them to be distinguished from viral RNAs (Figure 1C; Nichols et al., 2021; Herbert, 2019b; Herbert, 2021). The Z-RNA tags are recognized by the Zα domain of ADAR p150 and enable the negative regulation of IFN responses against host RNAs (Figure 1D). Genetic evidence for this mechanism is provided by Aicardi-Goutières Syndrome type 6 (AGS6). Mutations that lead to a loss of ADAR p150 binding to ZNA are causal for this interferonopathy. In AGS6, responses to self dsRNAs continue unabated (Herbert, 2020b; Guo et al., 2023; Liang et al., 2023).

What then triggers the flip of the ALU Z-BOX to Z-RNA? The IFN responses are activated by dsRNA sensors such as the helicase MDA5 (melanoma differentiation-associated protein 5, encoded by IFIH1, interferon induced with helicase C domain 1) that form filaments on any long A-RNA helix. The stretching of dsRNA by MDA5 as it coats the RNA causes tension that can be relieved by flipping the helix from right-handed A-RNA (pitch = 24.6 Å) to left-handed Z-RNA (pitch = 45.6 Å). The relaxation enables MDA5 to complete ATP hydrolysis, leading to dissociation of the filament and termination of the IFN response (Herbert, 2021). As Z-RNA forms, the IFN-induced ADAR1 p150 isoform that binds Z-RNA docks and can initiate A to I editing to trigger removal of self-RNAs by inosine-specific nucleases (Wu et al., 2019). The Z-BOX of EREs and ADAR p150 work as a tag team to protect the host against immune responses against self.

## Eliminating threats

Quite surprisingly, given the age of the Zα domain, there is only one other protein in the human genome, ZBP1 (Z-DNA binding protein 1). This protein can induce inflammatory cell death and serves to amplify anti-pathogen responses. Usually, activation of ZBP1 is squelched by ADAR1 binding through the masking of ZNAs (Zhang T. et al., 2022; Jiao et al., 2022; Hubbard et al., 2022; de Reuver et al., 2022). In normal cells, levels of ADAR1 p150 and ZBP1 are sufficient to maintain homeostasis. However, when IFN responses persist, as happens during viral infection, there is a more than a hundred-fold induction of ZBP1 protein that overcomes any ADAR1 p150 suppression. The IFN induced transcription of the normally silent, Z-RNA forming, EREs then triggers ZBP1 activation. ZBP1 then terminates the threat that triggered the IFN alarm.

Usually the ZBP1-dependent response results in necroptosis, a form of inflammatory programmed cell death (PCD) that activates the acquired immune system. The layered defense amplifies antigen-specific T and B cell memory subsets to protect against future attacks by the pathogen and its relatives. The strategy is quite general. The innate immune response based on host dsRNAs is built in. It does not depend on prior exposure to a pathogen or on whether the threat is old or a newly emergent one. There is no need for a pathogen-specific gRNA. The same process as used to defend against viruses is able to induce adaptive immune responses against dysfunctional and malignant cells. In both cases, the abnormal peptides expressed by host cells target them for elimination by the adaptive immune system, ending the threat. With this strategy, defense is not based on sequence-specific gRNAs. Instead, the response is initiated by ERE-derived dsRNAs with immunity provided by antigen-specific proteins.

Like many other cells, neurons do not normally express ZBP1 (Solon et al., 2024), so they do not usually undergo PCD when ZNAs accumulate in response to elevated IFN levels. However, other brain cells express ZBP1 and are eliminated in this manner. ZBP1 can also activate a different set of pathways to resolve the threat. For example, in response to ZIKA virus, ZBP1 increases expression of IRG1 (immune responsive gene1, encoded in humans by ACOD1, aconitate decarboxylase 1). The enzyme promotes formation of itaconate (Daniels et al., 2019), which leads to the nuclear translocation of TFEB (transcription factor EB) (Zhang Z. et al., 2022), the master transcriptional regulator of autophagy and lysosomal biogenesis. The endosomes induced in phagocytic cells direct viruses to lysosomes, an attempt to restrict their replication, one that is resisted by many pathogens (Campbell et al., 2015; Contreras et al., 2023; Chen et al., 2023; Zhai et al., 2023).

Not all effects of dsRNA depend on Z-RNA formation. Some are mediated by the dsRNA sensor toll-like receptor 3 (TLR3) that is activated by poly (I-C), an RNA duplex that has inosine on one strand paired to cytosine on the other, but does not form Z-RNA. Administration of the dsRNA polymer to animals of age 4 to 9 months induces TLR3 responses that are protective in the APPswe/PSEN1dE9 transgenic mouse model of Alzheimer’s disease (AD) (Wang et al., 2023). Activation of NF-κB (nuclear factor kappa B subunit 1 encoded by NFKB1) that promoted clearance of amyloid was reported but not by induction of IFN or through inflammatory responses. In a separate study, TLR3s activation was prevented by binding of APOE (apolipoprotein E) proteins, variants of which increase the risk of AD. APOE in this case is bound to the heparan sulfate proteoglycans (HSPG) that decorate cell surfaces (Zhu et al., 2010). Interestingly, another study revealed that the APOE4 variant that also confers the greatest risk for AD, binds with much higher affinity to HSPG than other variants. Binding was enhanced by the unusual 3-O-sulfo heparan sulfate (3-OS) modification that is made by the enzyme HS3ST1 (heparan sulfate-glucosamine 3-sulfotransferase 1) (Mah et al., 2023). Two well powered GWAS studies show that variants of HS3T1 gene with highest activity are likely associated with increased risk of AD (Witoelar et al., 2018; Jansen et al., 2019). Whether the binding of APOE4 to 3-OS also inhibits TLR3 activation by immune EREs is currently unknown. Potentially, the interaction provides an additional connection between immune EREs and memory. The non-neuronal cells activated by dsRNA through TLR3 then protect neurons from danger by removing threats, likely via phagocytosis.

## Neurological impact of ERE-induced immune responses

In AD and many other human neurological disorders, elevated levels of IFN stimulated transcripts and ERE encoded dsRNAs are present. Many of the outcomes have been attributed previously to oxidative stress from either mitochondrial damage or unfolded protein responses (Larsen et al., 2018; Cheng et al., 2021; Ravel-Godreuil et al., 2021). Besides effects of EREs on innate immune responses in the brain, the induction of chronic IFN also directly impacts neurological memory. This outcome is observed in mouse models of immunodeficiency that impair adaptive immune responses and are characterized by the emergence of endogenous, ecotropic murine leukemia viruses. In a B cell immunodeficiency strain produced by knockout of the immunoglobulin heavy constant mu gene (muM mice), there is cognitive impairment of hippocampus-dependent learning tasks. The deficits are resolved by knockout of MDA5, the cytoplasmic dsRNA sensor that induces IFN responses. Both contextual fear memory (CFM), and spatial learning tasks are improved in animals when both muM and MDA5 coding sequences are absent (Sankowski et al., 2019). Impaired spatial memory is also observed in animals with severe combined immune deficiency (SCID) and in thymic, T cell deficient nude mice. Collectively, the results connect immune and memory EREs (Kipnis et al., 2004).

A separate report provided independent evidence that increased dsRNA plays an essential role in conditioned fear extinction learning (Marshall et al., 2020). In this study, ADAR1 expression in the infralimbic prefrontal cortex was knocked down using short hairpin RNAs. The responses were restored by transduction of ADAR p150 constructs with a wildtype Zα domain, but not by expression of ADAR p110 or ADAR p150 variants that had loss of function mutations in either the Zα or deaminase domain. RNA editing activity was required for fear extinction to occur. The edited RNAs were enriched for postsynaptic membrane, synapse, postsynaptic density, and dendritic spines gene ontology terms. As well as potentially recoding exons, the adenosine to inosine editing likely promoted triage of dsRNAs by inosine-specific nucleases. The editing sites and ADAR binding sites were closely approximated to EREs. Together the results from the studies of ADAR1 p150 transduction and MDA5 knockout immunodeficient mice suggest a different model for long-term impairment of brain function that is observed with chronic viral infection and in neurodegenerative diseases. In these cases, the failure to clear EREs leads to high interferon levels and a reduced ability to form new memories. How then do immune EREs coordinate or interfere with memory EREs?

## ERE and neural memory

The increased expression of immune ERE triggers responses to protect the host against viruses and other threats. The evidence suggests that these responses negatively impact the long-term potentiation of memory formation. The findings raise many questions: what is the mechanism involved in cognitive impairment and how does this relate to the enhancement of neuronal plasticity by memory EREs such as ARC? The coevolution of LTR GAGs and non-LTR transcripts raises the possibility that SINEs and other ncRNAs can interfere with the transmission of ribotransmitters between neurons that could also potentially impact neuroplasticity, as in the case of ARC. Further, a class of immune EREs could retain the UTR required for packaging the ERE transcripts into a particular GAG capsid, similar to the way RNA is assembled into PEG10 VLC (Segel et al., 2021). Transmission of immune and memory ERE would then be regulated by expression of those VLC and the RNAs they engage.

Immune ERE potentially could impact cellular function on either side of the synapse through their effects on the translation of ribotransmitters like *ARC*. The effects on the presynaptic side could be via phosphorylation of EIF2α by the IFN induced dsRNA activation of PKR (Kaempfer, 2006; Halliday and Mallucci, 2014). Phosphorylated-EIF2α (phosEIF2α) plays an important regulatory role in neuronal plasticity. It acts by increasing the translation of ATF4 (activating transcription factor 4), a protein that impairs memory by repressing expression of CREB (cAMP responsive element binding protein), an important mediator of L-LTP (long-long-term potentiation) and LTM (long-term memory) (Costa-Mattioli et al., 2007). Notably, PKR loss of function *Eif2ak2^Δ12/Δ12^* mice decrease phosEIF2α levels and have enhanced memory formation. The improved LTM results from a reduction in GABAergic inhibitory postsynaptic currents (IPSC) due to decreased presynaptic levels of phosEIF2α, with no evident effects on excitatory postsynaptic currents (Zhu et al., 2011).

The *Eif2ak2* exon 12 deletion model used in this study removes the PKR catalytic domain (Zhu et al., 2011). The knockout does not affect brain morphology or alter the axonal or synaptic histological markers tested. However, the PKR variant is not, as originally thought, a null allele: the gene still expresses a truncated version of PKR with an intact N-terminal dsRNA binding domain and is able to scaffold both IFN and NF-κB responses (Baltzis et al., 2002). The outcomes are due solely to the loss of the kinase activity. The result is supported by a gain of function mouse model where the PKR kinase is triggered by using AP20187 to dimerize and activate a PKR allele fused to the FK506 binding protein. By increasing phospEIF2α levels, it was possible to promote long term depression (LTD) by reducing the efficacy of synaptic transmission. Unlike LTP, the establishment of LTD required protein synthesis (Di Prisco et al., 2014). Similarly, cognitive function was impaired in wild-type mice treated with IFN. The treatment increased expression of both immune ERE and *Eif2ak2* transcripts, resulting in PKR activation and EIF2α phosphorylation (Zhu et al., 2011). Collectively, the results link the modulation of translation by immune ERE to LTP and LTD.

*ARC* mRNA translation is also impacted by the phosphorylation status of EIF2α. Normally, EIF2α promotes the 7-methylguanosine CAP-dependent translation of *ARC* mRNA. Following synthesis, the protein is assembled into capsids, which then convey *ARC* mRNA from the presynaptic boutons to postsynaptic structures, such as those present on muscle cells and dendritic spines (Epstein and Finkbeiner, 2018; Ashley et al., 2018; Pastuzyn et al., 2018). The translated *ARC* mRNA modulates the internalization of AMPA receptors through interactions with proteins like endophilin-3 and dynamin-2 that modulate clathrin-mediated endocytosis. The effects likely remain localized to the spine in contact with the bouton. Indeed, live cell imaging reveals that the mixing of the spine contents with those of the cell body is restricted by the constriction at the base of the spine (reviewed in Adrian et al., 2014). Further, effects of activation induced translation are likely more pronounced in splines as the change in ion concentrations following depolarization are magnified due to their small volume (reviewed in Dastidar and Nair, 2022). The design allows the targeted reprogramming of individual synaptic connections between pre- and postsynaptic cells.

During viral infection, the phosphorylation of EIF2α by PKR inhibits the normal path of CAP-dependent translation of transcripts. Instead, the synthesis of ARC proteins depends on the initiation factor EIF4G2 that enables non-CAP dependent translation though an internal ribosome entry site (IRES) (Pinkstaff et al., 2001; Thakor and Holcik, 2012; Hacisuleyman et al., 2024). Besides ARC, EIF4G2 permits translation of other neuronal proteins in neurons that promote local dendritic remodeling and disruption of synaptic contacts (David et al., 2022; Shestakova et al., 2023; Hacisuleyman et al., 2024). Indeed, the disassembly of key structural components in dendritic spines is triggered by herpes simplex virus (HSV) type I infection of neurons (Acuna-Hinrichsen et al., 2021). Further activation of PKR protects against endoplasmic stress that can otherwise induce neural apoptosis and necroptosis (Yuan et al., 2019). So far, there is no published data on HSV infection of *Arc* deficient mice. Such a study would inform on the role of ARC protein in the transmission of immune EREs. Interestingly, in traumatic brain models, knockdown of ARC protein increases neuronal cell death from apoptosis and necrosis (Chen et al., 2020). While the effects described are usually interpreted as due to the effects of ARC on intracellular signaling (Chen et al., 2024), the transmission of ribotransmitters across the synapse by VLC could further protect bystander cells to limit neuronal loss.

## VLC and EREs as an antiviral defense

The findings raise the question of whether the ERE encoded immune RNAs and VLC-forming GAG proteins act together to defend against viral infections of the nervous system. The simplest model is that during viral infections, “junk” dsRNAs produced by immune EREs are transported by VLC across the synapse to induce an antiviral state in the recipient cell through the activation of PKR. The process alerts postsynaptic neurons of the threat posed by the virus. The PKR response then restricts the translation of infectious agents transmitted from the presynaptic cells (Taghavi et al., 2013). The defensive strategy is not perfect. This early warning system will also alter the programming of new memories by shutting down the EIF2α dependent translation of the coterie of mRNAs required for strengthening synaptic communication. However, the strategy has major benefits. The effect is localized. Only actively connected synapses are targeted. Further, the response is rapid: there is no need to wait on the recipient cells to mount an IFN-stimulated response to limit the spread of viruses. In extreme cases, these responses eliminate infected cells by inducing necroptosis, countering the protective effects of ADAR p150 and PKR.

If persistent over time, the elevated immune EREs and cell-intrinsic interferon responses may induce more serious neurological sequelae, as occurs in long COVID syndromes (Bungenberg et al., 2022). The chronic elevation of immune EREs within neurons in other types of neurodegenerative diseases may also aggravate the pathology in other ways. The phosphorylation of proteins by PKR other than EIF2α, such as tau protein (encoded by MAPT, microtubule associated protein tau) will also contribute to outcomes. The phosphorylation of tau interferes with its normal role in synaptic vesicle recycling (Hori et al., 2022; Jaye et al., 2024). This effect is likely beneficial during infection. It will inhibit the release and uptake by neurons of viral particles, thus preventing the spread of pathogens by retrograde transport. However, the response will also impair the clearance of protein aggregates from cells, such as those formed by phosphorylated tau. Consistent with this outcome, tau protein modified at sites phosphorylated by PKR accumulates in tauopathies, AD and in other neurodegenerative diseases (Cavallini et al., 2013; Sossin and Costa-Mattioli, 2019; Reimer et al., 2021; Martinez et al., 2021). One of these modifications produced by PKR (and other kinases) results in phosphorylation of tau at threonine 181 (ptau118). Plasma levels of ptau118 predict the presence of amyloid beta (Aβ) deposits in a number of different brain regions linking PKR activation to disease outcomes (McGrath et al., 2022).

## Therapies

How then can we address the potential problems arising from immune ERE suppression of LTP and LCM associated with neurodegenerative disease? Since prevention is preferable to intervention, a focus on decreasing the IFN-induced expression of EREs and decreasing phosEIF2α levels are intriguing approaches. Many studies have established a relationship between PKR activation and disease. In one cohort, PKR levels were elevated in the serum of 17 AD patients compared to 27 unaffected controls and another 19 patients with mild cognitive impairment (Monllor et al., 2021). A similar finding was reported by a different group that found elevated PKR levels in the CSF (cerebral spinal fluid) of 45 AD patients compared to 11 patients with mild cognitive impairment and 35 unaffected controls (Mouton-Liger et al., 2012). Further, CSF levels of phosphorylated PKR positively correlated with those of ptau118 (Mouton-Liger et al., 2012). The potential use of ptau118 as a prospective plasma biomarker for future increased Aβ deposition across multiple brain regions was validated using the Framingham Heart Study cohort (McGrath et al., 2022). Collectively these findings and those described earlier favor selective therapeutic targeting of the PKR catalytic activity, an approach that would leave intact other kinases that promote clearance of amyloid and tau proteins via the UPR. Older PKR inhibitors and current ones like C16 appear to lack sufficient specificity for clinical deployment (Chen et al., 2008; Lo et al., 2019). Newer PKR antagonists are in development (Lopez-Grancha et al., 2021).

Therapeutic strategies aimed at reducing the dsRNA in neurons and thereby reducing PKR activation are less certain. Potentially such approaches could prevent the reactivation of autonomously replicating EREs. Of note is the real-world data analysis that reports a decreased risk of AD in patients prescribed reverse transcriptase inhibitors, a finding validated in the preclinical studies so far performed (Magagnoli et al., 2023). A different strategy focuses on therapeutics that reverse the epigenetic changes associated with an increased expression of ERE (Cao et al., 2022). Unfortunately, the current generation of epigenetic therapeutics have shown greater toxicity than expected (Feehley et al., 2023). Another approach advocates the intranasal administration of IFN (Azarafrouz et al., 2022). However, the manipulation of interferon levels to reduce ERE expression is complicated. IFN-based treatments are reported to promote the autophagy of protein aggregates, yet the same processes also rapidly down-regulate IFN receptors (Tian et al., 2019). Further, *Ifng*^–/–^ knockout mice that do not express type 2 IFN, have enhanced hippocampal spatial learning and novel object recognition, suggesting that stripping this receptor from cell surfaces in the brain may be preferred (Monteiro et al., 2016).

EREs may be of interest as biomarkers. The correlation of cognitive impairment with GAG encoded mRNAs and SINE levels in blood could be useful as a surrogate for ongoing memory impairment and for assessment of treatment responses, especially those transcripts that are associated with ARC and other GAG encoded VLCs. The mRNAs conveyed by these capsids may also provide clues for designing low molecular weight, brain-permeable therapeutics that enhance memory consolidation by either supplying the ribotransmitters or the proteins they encode or that enhance their actions.

## Conclusion

Here, I have focused on the potential for reprogramming of postsynaptic dendritic spines by ribotransmitters conveyed by VLCs from pre- to postsynaptic neurons. Transmission by the ARC VLC *ARC* mRNA exemplifies this mechanism. The information delivered from the broadcasting bouton to the recipient cell depends on the current state of both. The exchanges tweak responses to future incoming messages. The optimal pairing requires the upstream neuron to send and receive the correct set of information and exploits the phenotypic plasticity of dentate gyrus granule cells (DGGC). The game of baseball provides an analogy: the presynaptic neuron has the role of a pitcher, with the downstream neuron signaling the type of delivery expected by the catcher. This feed-forward arrangement ensures that both pitcher and catcher are copacetic: it ensures that there is a high degree of synchronicity in their actions. The different pitches correspond to the range of inhibitory and excitatory neurotransmitters expressed by DGGC, but the downstream catcher only expects just one of them at a time. The catcher’s response will signal to the pitcher whether the requested ball was thrown. The pitcher will also learn what type of pitches the catcher can handle.

Just as the mix of pitches in baseball determine the outcome of the game, the balancing of LTP and LTD responses enabled by ribotransmitters and receptors of the presynaptic neuron underlies the adaptive memories created (Zhu et al., 2011; Mody, 2002; Gutierrez, 2016). Just as teams over time will choose pitchers that work best with the catchers that they have available, natural selection can optimize the ability of the presynaptic neuron to send and receive the signals that optimize outcomes. Infectious agents exploit the vulnerabilities inherent in the use of memory EREs and ribotransmitters to exchange information across synapses via VLC transported by extracellular vesicles, just as an opposing team might learn the catcher’s signals to change the outcome of a game. Such threats are countered by immune EREs that thwart autonomous replicants by immediately inhibiting the translation of their messages in the recipient cell, preventing the hostile takeover of the postsynaptic neuron. The exaptation of ERE proteins and RNAs enhance the ability of a species to reproduce. The coevolution of memory and immune EREs frame the flourishes and functionalities of future generations.

The review highlights the complex role of EREs in mammalian evolution. Memory EREs contribute to synaptic complexity through the capsids they encode and the ribotransmitters they transport. Immune EREs form dsRNAs that activate antiviral defenses to impede the spread of infectious agents from one neuron to the next. Both sets of ERE impact translation through different initiation factors: EIF2α promotes LTP by cap-dependent translation, while dsRNA activation of PKR produces LTD by the non-CAP mediated translation of a different subset of mRNAs. The balance between LTP and LTD changes according to context and is perturbed during disease. The memory EREs promote neuronal plasticity while the immune EREs are SINEs of forgetfulness.

## Data Availability

The original contributions presented in the study are included in the article/supplementary material, further inquiries can be directed to the corresponding author.
